# Perceived interpersonal distance changes in young Taiwanese pre and post SARS-CoV-2 pandemic

**DOI:** 10.1038/s41598-024-51278-2

**Published:** 2024-01-05

**Authors:** Yi-Lang Chen, Yu-Chi Lee, Che-Wei Hsu, Andi Rahman

**Affiliations:** 1 Department of Industrial Engineering and Management, Ming Chi University of Technology, 84 Gung-Juan Road, Taishan, New Taipei, 243303 Taiwan; 2 Department of Industrial Engineering and Management, National Taipei University of Technology, Taipei, 106344 Taiwan; 3 Department of Industrial Engineering, Andalas University, Padang, 25175 Indonesia

**Keywords:** Psychology, Health care

## Abstract

The persistent SARS-CoV-2 pandemic, spanning over three years, has profoundly impacted daily life worldwide. Crucial measures like interpersonal distance (IPD) and mask-wearing have become paramount in preventing infection. With SARS-CoV-2 now resembling an endemic condition similar to influenza, it is vital to assess the changes in IPD influenced by relevant factors during and after the pandemic. This study concentrated on two specific stages (the pandemic stage and the post-pandemic era) and investigated variations in IPD with different test combinations. Variables taken into account encompassed the pandemic stage, participant gender, target gender, and mask-wearing status. We examined IPD data from 100 young individuals (50 males and 50 females) at each stage, with a one-year interval between tests. The results highlighted the substantial impact of all variables on perceived IPD during the pandemic phase (all *p* < 0.001). However, in the post-pandemic stage, only mask-wearing demonstrated a notable effect on IPD (*p* < 0.001). As the SARS-CoV-2 epidemic subsides, the enduring influence of mask usage on IPD persists. Nevertheless, the gap between the two mask-wearing scenarios diminishes, contracting from around 50 cm during the epidemic phase to 20 cm in the post-epidemic phase. Across these two pandemic stages, there was an overall reduction of approximately 90 cm in IPD, indicating a noteworthy decrease in perceived personal space and a consequential shortening of social proximity during the post-pandemic stage. This decrease in IPD may suggest the successful socio-cultural adaptation of the young Taiwanese individuals in our study during the post-pandemic era.

## Introduction

In recent years, the SARS-CoV-2 pandemic, along with government guidelines aimed at curtailing its transmission, has significantly altered our daily lives. The significant threat to human life and the global challenge facing public health strategies have led governments worldwide to implement strict preventive measures. Among these measures, the most widespread and effective involve establishing social distancing norms and enforcing mask-wearing regulations. These actions primarily aim to minimize the risk of cross-infection between individuals^1,2^. Notably, these policies have played a crucial role in successfully curbing the further spread of the epidemic, contributing significantly to its global alleviation and control. During this period, the concept of interpersonal distance (IPD) has emerged as a prominent focus of research^2–4^. The variations in IPD across diverse variables and situations also have significant implications for individuals' social lives and interpersonal relationships.

Throughout the SARS-CoV-2 pandemic, the concept of social distancing has entered everyday conversations^5,6^. Governments have implemented policies to promote physical distancing as a strategy to diminish virus transmission. The World Health Organization advocates various established methods to prevent SARS-CoV-2 transmission, including regular handwashing, vaccination, adhering to social distance, and wearing masks ^7^, especially in public spaces^8^. The mandate to uphold a 1.5-m distance from others in public areas is based on the recognition that respiratory viruses, such as coronaviruses and influenza, primarily propagate through the inhalation of respiratory droplets^1^.

Prior research on IPD during past epidemics, has indicated that maintaining a distance of over 1 m can significantly reduce the risk of infection^9^, and the use of surgical masks has been recommended^10^. Despite the documented effectiveness of these measures in preventing transmission^9,11^, policies involving social distancing and isolation may have psychological implications^12,13^. For instance, wearing face masks conceals facial expressions, which can influence people's emotions and cognitive processes during social interactions^3,14^. People around the world have adapted to wearing face masks and adhering to recommended IPDs in their everyday routines, leading to immediate consequences like discomfort, heightened alertness, and diminished social cues^15^. Additionally, individuals' vaccination status significantly affects IPD, although vaccination does not guarantee reduced infectivity from person to person^8^. Research conducted in Germany and Italy has shown variations in IPD correlated with the incidence of SARS-CoV-2^6,16^, and different types of social interactions also influence IPD preferences^17^. Nevertheless, the long-term consequences of deviating from preferred IPD norms, particularly in the post-SARS-CoV-2 era, remain uncertain.

In 2003, Taiwan emerged as one of the areas most impacted by SARS, but in the initial year of the SARS-CoV-2 pandemic^18,19^, it stood out as one of the least affected locations—particularly in comparison to neighboring countries^20^. Survey analysis by Pandey and Yu^21^ revealed notably positive experiences among foreign residents in Taiwan during this period. These residents expressed comfort and a sense of safety, crediting successful policies for preventing community outbreaks. Additionally, the high level of adherence to mask-wearing and social distancing regulations among the public played a crucial role in this positive outcome. As the epidemic begins to show signs of subsiding, several countries are gradually easing their strict policies. In Taiwan, for instance, the mandatory mask-wearing requirement was lifted on April 17, 2023, indicating a diminished virus threat in various regions. Simultaneously, the daily announcement of confirmed cases ceased. However, a significant question arises: How do individuals, who have become accustomed to wearing masks and adhering to appropriate IPD in recent years, perceive these anti-epidemic behaviors in the post-pandemic era? This pertains particularly to the re-establishment of personal social relationships and the reassessment of personal safety awareness.

To address the aforementioned question, our study aimed to collect IPD data during two distinct stages of the epidemic in Taiwan. We endeavored to compare the alterations in IPD under various conditions, considering the variables under investigation. We scrutinized the differences between the peak of confirmed cases (May 2022) and the period subsequent to the removal of mask-wearing regulations (April 2023) by enlisting 100 participants to provide IPD data for each of these periods. The independent variables fall into three categories: participant gender (male and female), target gender (male or female), and target mask-wearing status (wearing or not wearing masks). From a proxemic perspective, we hypothesize that IPD may adapt asymmetrically over time^16^. Welsch and colleagues^16^ noted that the favored IPD quickly adjusted in response to distance requirements, yet an expansion of IPD may persist to some extent after the SARS-CoV-2 pandemic crisis. This suggests we can expect a rapid increase in the preferred IPD during the peak of the pandemic, while the preferred IPD may decrease at a relatively slower pace after the pandemic. Our study hypothesis posits that in the post-pandemic period, there may be an expected decrease in IPD. However, various variables could exert distinct impacts on IPD.

## Materials and methods

### Participants

During the peak of the epidemic in Taiwan in May 2022, we conducted an experiment involving a total of 100 participants, equally divided between males and females (50 each). Data collection took place from May to June 2022, aligning with the peak of confirmed cases recorded on May 27, 2022, totaling 94,808. About a year later, a comparable experiment was replicated, and IPD data were collected from April to May 2023. Importantly, the test was conducted subsequent to the government's announcement on April 17, 2023, that wearing masks would no longer be mandatory. In total, we recruited 200 participants, with 100 individuals contributing IPD data for each of these periods. Due to some participants exiting the study for various personal reasons, approximately one-third of the participants were duplicates. All participants held either undergraduate or graduate degrees and reported no cognitive or psychological issues.

In the 2022 test, the average (standard deviation) ages for male and female participants were 20.8 (1.8) and 20.7 (1.6) years, respectively, with all participants having received at least one dose of the SARS-CoV-2 vaccine. The vaccination rate for the second dose was 56%. In the 2023 test, the corresponding average (standard deviation) ages were 20.6 (1.7) and 20.8 (1.8) years, respectively. In this stage, all participants reported receiving two doses of the vaccine, with 86% of them having completed the third dose. All participants were right-handed for mouse operation and were not previously familiar with the targets in the experiment. Informed consent was obtained from all participants and attested for publication of the identifying information/images in an online open-access publication. The study obtained approval from the Ethics Committee of Chang Gung University, Taiwan, and all methods were carried out in accordance with the relevant guidelines and regulations of the 2013 World Medical Association Declaration of Helsinki. Informed consent was obtained from all participants.

### Experimental setting

Due to the SARS-CoV-2 pandemic, we chose for an online survey method to collect IPD data in the initial test stage in 2022, adhering to recommended precautions to prevent human-to-human transmission^22,23^. To ensure consistency and facilitate comparison, the second test stage in 2023 was also conducted using the same online survey format. This online survey was adapted from the paper-and-pencil test utilized in studies by Hayduk^24^ and Xiong et al.^25^. Online surveys have proven to be effective tools for collecting IPD data and are widely employed in clinical and practical research^26^. The survey was administered using a computer with the Axure RP rapid prototyping tool (Axure Software Solutions, San Diego, CA, USA).

During the test, participants were instructed to use the cursor to manipulate a virtual subject (avatar) toward a target. To prevent any influence on the participant's distance judgment, the arrow indicating the movement direction between the two avatars was concealed when participants initiated the avatar's movement. Essentially, no visual cues were presented regarding the distance between the two avatars during the determination of IPD. Participants were tasked with visualizing and determining the IPD by positioning the avatar at a location where it remained comfortable but had just begun to feel uncomfortable. This definition of IPD is consistent with that employed in prior studies^8,24,27–29^. The distance between the avatars was then adjusted, maintaining a 1:7.2 ratio, to derive the psychological IPD. Initially, the two avatars were separated by a distance of 55.5 cm, corresponding to approximately 4 m in real-world terms between the participant and the target^8,29^. The measurement’s reliability was assessed through a pilot study, and the results indicated a satisfactory intraclass correlation coefficient of 0.85, confirming its reliability.

### Targets

As targets for the study, we selected two individuals, a 22-year-old man and a 22-year-old woman, who displayed typical Chinese features. The man had a height of 176 cm, while the woman stood at 160 cm, and and both targets wore everyday clothing without additional accessories. To generate digital targets for the online survey, we employed a digital camera (Sony HDR-XR260; Sony, Tokyo, Japan) to capture frontal images of these individuals under two distinct mask-wearing conditions. The targets were directed to maintain neutral expressions during the image capture process. These captured images were later incorporated into the online survey. On the screen, the heights of the digital male and female targets were adjusted to 24.4 cm and 22.2 cm, respectively. The surgical masks employed in the study were standard plain blue masks without any decorative elements, consistent with the type of face mask typically recommended during the SARS-CoV-2 pandemic.

### Procedure and design

The test procedures for both the initial assessment in 2022 and the second assessment in 2023 were conducted with high consistency. Prior to commencing data collection for each test, a research facilitator provided participants with a detailed explanation of the testing process. Additionally, to evoke a recollection of their experiences during the SARS-CoV-2 pandemic, a 2.5-min video produced by Stanford Medicine was presented to the participants. The video primarily, through animation, illustrates the transmission of SARS-CoV-2 among people and how transmission can be prevented. To enhance the quality of IPD data collected, we displayed four images of the targets in 2 × 2 combinations corresponding to the specific conditions just before participants made their judgments regarding perceptual distance. These images were intended to assist participants in immersing themselves in the scenario and imagining the experience of facing the target under various circumstances.

During the test, each participant was instructed to complete three separate trials, and subsequently, the average values of these trials were computed for further analysis. To mitigate participant fatigue, a mandatory minimum 2-min rest period was allocated to each participant between consecutive trials. The trials were presented in a sequential manner for IPD judgments and were randomized in their order. In the IPD assessment, participants utilized a computer mouse to manipulate an avatar's position, choosing a location that they deemed psychologically close to becoming uncomfortable but still within the boundaries of comfort. Participants had the flexibility to make slight adjustments to the avatar's position to confirm their perceived distance. Once participants had finalized their IPD, the computer automatically calculated and recorded the distance between the chins of the two avatars, following the procedures outlined in Lee and Chen^2^. Consequently, we amassed a total of 2,400 data samples, encompassing two test periods, each involving 100 participants, and covering two target genders, two mask-wearing conditions, and three repetitions.

### Statistical analysis

To collect and compare IPD data at two stages of the epidemic in Taiwan and identify the influences of various investigated variables on IPD, this study included four independent variables: the test period, participant gender, target gender, and face mask wearing. The primary dependent variable under investigation was the IPD, expressed in cm. The statistical analysis was performed using SPSS 23.0 (IBM, Armonk, NY, USA), with a predefined significance level (α) of 0.05. To evaluate the impact of these independent variables on IPD, a four-way analysis of variance (ANOVA) was carried out. In this analysis, the pandemic stage and participant gender were categorized as between-subject factors, while the remaining variables were considered within-subject factors. Additionally, two distinct three-way ANOVAs were conducted for each test period, followed by post hoc comparisons employing independent t-tests. Effect sizes were quantified using η^2^ values for each effect, as outlined by Cohen^30^. To ensure the robustness of the analysis, the Kolmogorov–Smirnov test^31^ was employed to assess the normal distribution of numerical variables, and Levene's test^32^ was utilized to gauge the homogeneity of variances.

## Results

### Four-way ANOVA result and its interaction effects

**Figure 1:**
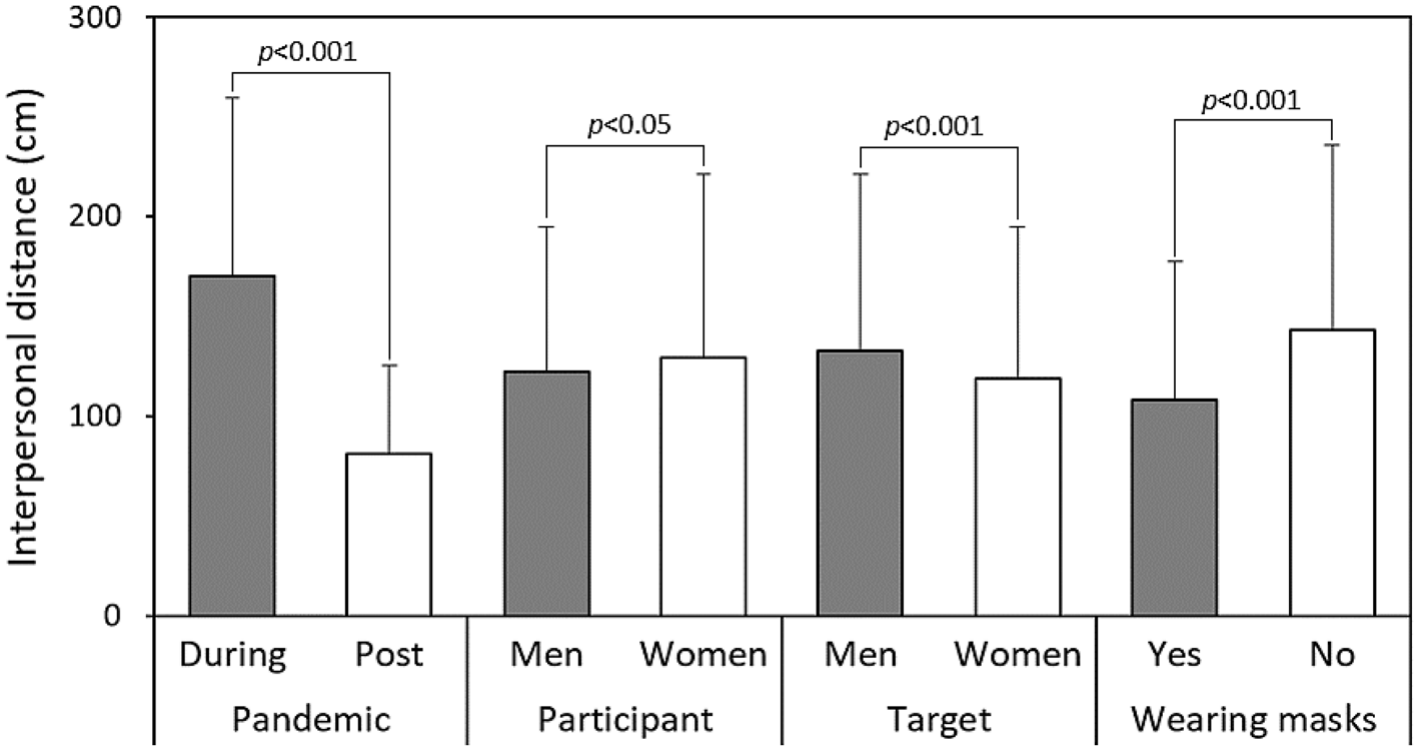
Comparisons illustrating the main effects of the four independent variables.

Table 1 presents the results of the four-way ANOVA for IPD measurements, indicating that all independent variables significantly influenced IPD (participant gender showed *p* < 0.05, while all other variables showed *p* < 0.001). Figure 1 further provides a comparison of the main effects for the four independent variables, along with the results of independent t-tests. During the pandemic stage, female participants exhibited a relatively larger IPD (129.0 cm), or when the participant facing a male target (132.5 cm), especially when the target was not wearing a mask (142.7 cm). In contrast, the corresponding IPDs were 122.0 cm, 118.7 cm, and 108.6 cm, respectively. This significant variation in IPD equated to an approximate 90 cm difference between the two pandemic stages (Fig. 1).

**Table 1:** Results of the four-way ANOVA for interpersonal distance.

Sources	SS	df	MS	F	Significance	η^2^
Stage (S)	3,180,701	1	3,180,701	699.95	< 0.001	0.306
Participant gender (PG)	19,586	1	19,586	4.31	< 0.05	0.009
Target gender (TG)	76,824	1	76,824	16.91	< 0.001	0.011
Mask (M)	464,904	1	464,904	102.31	< 0.001	0.061
S x PG	103,789	1	103,789	22.84	< 0.001	0.014
S x TG	40,282	1	40,282	8.87	< 0.01	0.010
S x M	90,474	1	90,474	19.91	< 0.001	0.012
PG x TG	28,197	1	28,197	5.10	0.074	0.003
PG x M	3571	1	3571	0.79	0.375	< 0.001
TG x M	2193	1	2193	0.48	0.487	< 0.001
S x PG x TG	835	1	835	0.18	0.668	< 0.001
S x PG x M	4142	1	4142	0.91	0.340	0.001
S x TG x M	1789	1	1789	0.39	0.530	< 0.001
PG x TG x M	629	1	629	0.14	0.710	< 0.001
S x PG x TG x M	87	1	87	0.02	0.890	< 0.001

### Three-way ANOVA results for each stage

As indicated in the ANOVA results presented in Table 1, the pandemic stage interacts with other variables, necessitating cross-analysis to delve into these interactions. Table 2 further elucidates the outcomes of the three-way ANOVA for each pandemic stage. The significant effects of both participant and target genders on IPD observed during the pandemic became non-significant in the post-pandemic stage. Although there was a statistically significant interaction effect between participant and target genders in the post-pandemic stage, its impact can be considered negligible due to the small η^2^ value.

**Table 2:** Results of the three-way ANOVA on interpersonal distance for each test stage.

Sources	During pandemic stage			Post-pandemic stage		
	F	Significance	η^2^	F	Significance	η^2^
Participant gender (PG)	14.86	< 0.001	0.018	3.20	0.152	0.004
Target gender (TG)	15.89	< 0.001	0.020	1.54	0.215	0.002
Mask (M)	67.18	< 0.001	0.078	38.16	< 0.001	0.046
PG x TG	2.70	0.101	0.003	5.08	< 0.05	0.004
PG x M	1.07	0.301	0.001	< 0.01	0.941	< 0.001
TG x M	0.55	0.457	0.001	< 0.01	0.941	< 0.001
PG x TG x M	0.08	0.774	< 0.001	0.07	0.798	< 0.001

**Figure 2:**
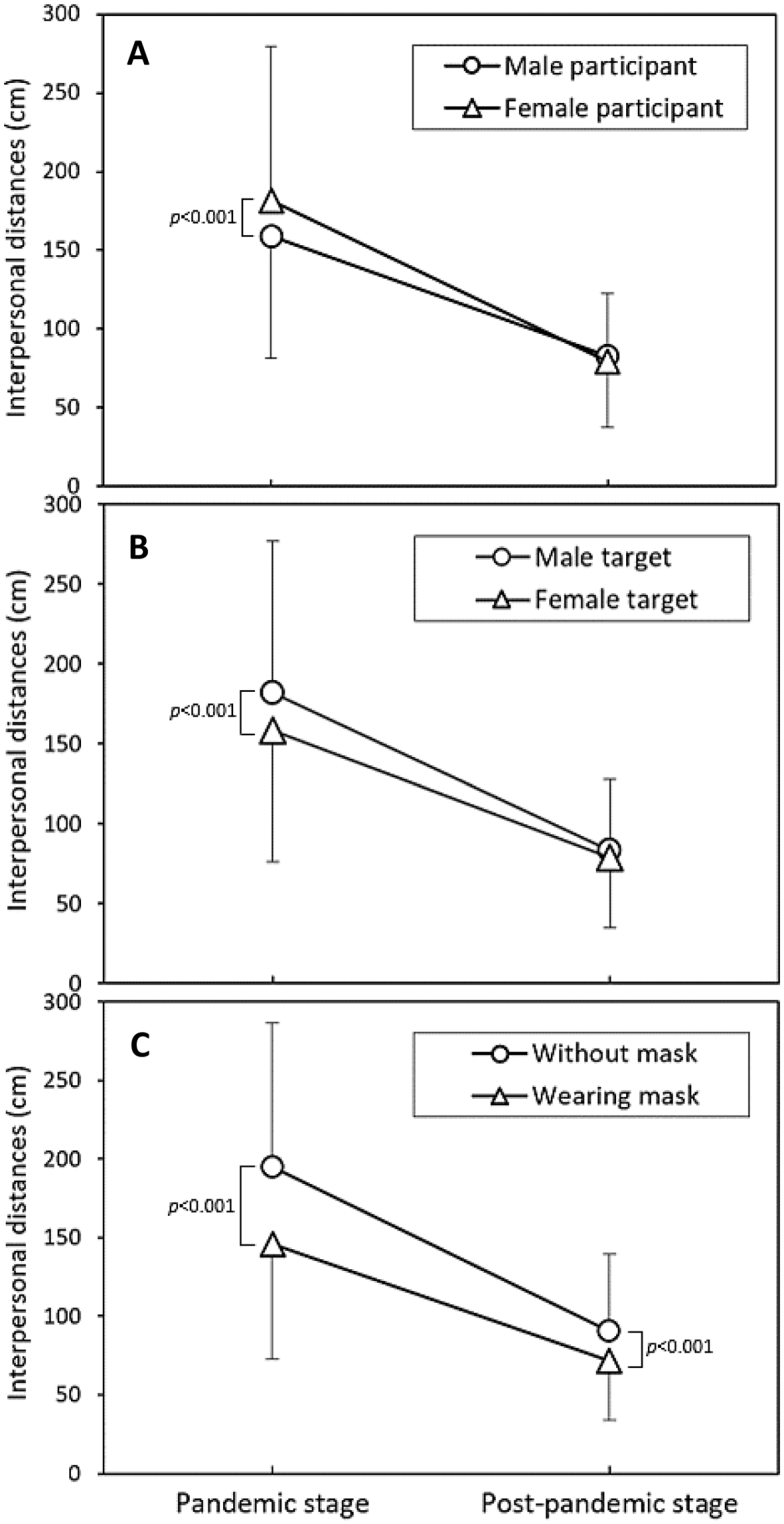
Illustration of the interactive effects of each independent variable with varying pandemic stages.

**Figure 3:**
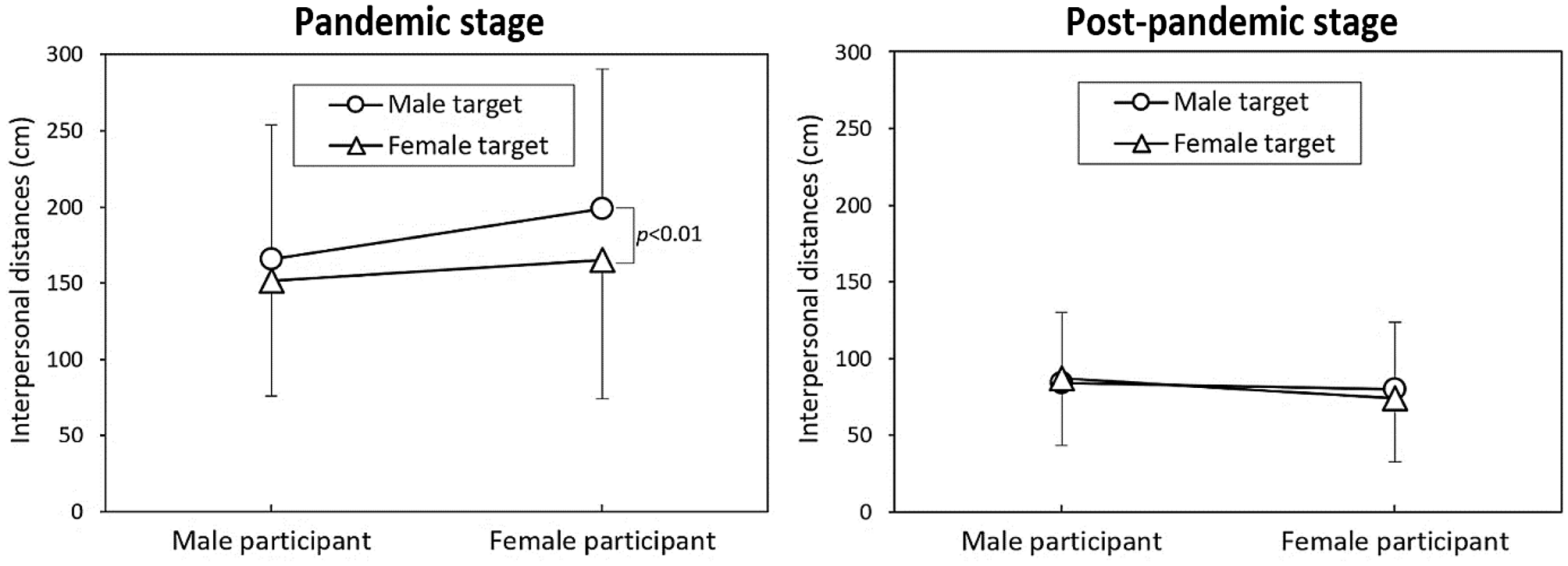
Differences in IPD under varying dyads combinations for the two pandemic stages.

Figure 2 depicts the interactive effects of the pandemic stage with participant gender (Fig. 2A), target gender (Fig. 2B), and mask-wearing status (Fig. 2C) on IPD. As depicted in the figures, female participants exhibited larger IPD, and male targets had a larger IPD during the pandemic stage. Conversely, during the post-pandemic stage, differences in IPD between paired levels converged, with both participant and target genders showing no significant differences in IPD. However, wearing masks at both stages had significant effects on IPD (Fig. 2C). In the analyses, the most notable differences in IPD between the two stages were observed in participant and target gender variables (Table 2). Figure 3 further illustrates the differences in IPD under various dyad combinations. The sole significant difference among the combinations was that female participants experienced a larger IPD when encountering male targets during the epidemic stage (*p* < 0.01).

### Paired comparisons between the two stages

**Figure 4:**
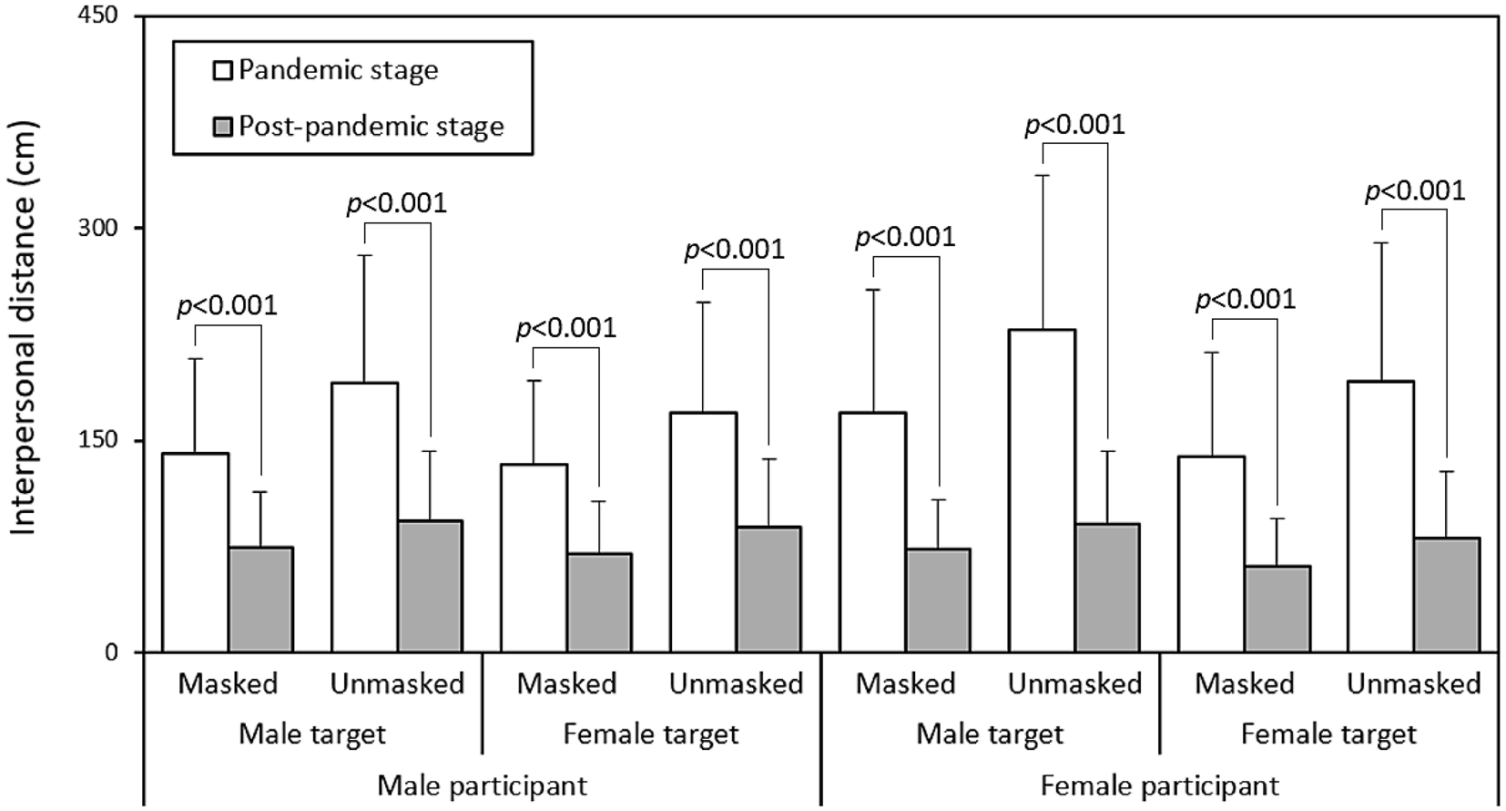
Pairwise comparisons in IPD for different test combinations between the two stages.

For a more visually accessible comparison of the differences in IPD between the two epidemic stages outlined in Table 2, Fig. 4 provides a detailed breakdown of each test combination, presenting the corresponding results analyzed through independent t-tests. In every distinct condition, all IPD values showed significant differences between the two pandemic stages (all *p* < 0.001).

## Discussion

During the SARS-CoV-2 pandemic, mask-wearing and maintaining interpersonal distancing became primary methods for self-protection. However, these preventive measures have had substantial impacts on people's daily lives and work. IPD is a crucial element in human communication and interaction. Therefore, as the pandemic comes to a close, people's longing for social interaction is reawakened, and their desire for connection grows stronger. Gaining insights into the changes in IPD can provide valuable information for shaping policies in the post-pandemic era. This study investigated the effects of different variables on IPD, and the ensuing impacts are discussed below.

### Pandemic stage

This study conducted two stages of online surveys to assess the impact of examined variables, including participant gender, target gender, and mask-wearing, on the perception of IPD during the SARS-CoV-2 pandemic and the subsequent post-pandemic stages. As anticipated, all independent variables significantly influenced IPD, with the most notable findings revealing interactions between the pandemic stages and the other three variables. Surprisingly, under identical experimental conditions, different pandemic stages resulted in substantial changes across all test combinations (Fig. 4). This underscores the significant impact of the pandemic on people's perceptions of personal space and social proximity.

When averaging across other variables, the IPD between the pandemic and post-pandemic stages significantly shortened, decreasing from 170.3 to 81.0 cm within a year, constituting nearly a 90 cm reduction. The field of proxemics, which studies human spatial behavior, has a history spanning over four decades^33^. Hall^33^ categorized interpersonal space into four distinct ranges: Intimate (0–46 cm), Personal (45–120 cm), Social (120–350 cm), and Public (> 350 cm). In our study, the results suggest that IPD has transitioned to the "Personal" range, even though the targets presented in the test were strangers to all participants. While we lack pre-pandemic IPD data for Taiwanese individuals, it is evident that 81.0 cm represents a relatively close distance for interactions between strangers. According to a previous survey on a global scale, the IPD of Southeast Asians towards social distance (strangers) and personal distance (acquaintances) before the epidemic was approximately 110 cm and 85 cm, respectively^34^. This suggests that the IPD of the young Taiwanese individuals observed in this study may have returned to, and possibly exceeded, pre-pandemic levels. However, when excluding the impact of masks, the associated IPD increased to 88.2 cm. This distance still appeared relatively short in comparison to the earlier findings of a multinational survey^34^.

Interestingly, Welsch et al.^16^ observed that people expected to maintain larger IPD from others even when there was no longer a risk of SARS-CoV-2 infection. Our study appears to have yielded contrasting outcomes. As indicated by Hall^33^, IPD appears to be influenced by various individual and cultural differences, and changes in IPD in the post-pandemic era may vary from one region to another, necessitating further investigation. Building on proxemic theory, Welsch et al.^16^ regarded IPD as an indicator of socio-cultural adaptation beyond the duration of the pandemic. The shortened IPD observed in our study may imply that these young Taiwanese individuals have exhibited exceptional socio-cultural adaptation in the post-pandemic era.

### Mixed-sex dyads

The results of our study clearly indicate that different pandemic stages have a significant impact on IPD. During the pandemic stage, all independent variables significantly influenced IPD (Table 2). Notably, female participants facing male targets without masks displayed relatively larger IPD, consistent with previous research conducted during the pandemic^2,3,6,8,35^. However, in the post-pandemic stage, only the mask-wearing variable continued to have a significant effect on IPD (Fig. 2C), while neither participant gender nor target gender appeared to impact IPD (Fig. 2A,B). This finding represents a noticeable departure from the results observed prior to the outbreak of the SARS-CoV-2 pandemic in 2019. Previous research on the effect of gender dyads on IPD has produced inconsistent results. Yu et al.^29^ found that male dyads reported the greatest IPDs, while female dyads reported the shortest IPDs, consistent with the patterns observed by Caplan and Goldman^36^ and Aliakbari et al.^37^. Conversely, Baxter^38^ and Evans and Howard^39^ found that the shortest IPD was observed in mixed-gender dyads, while Hecht et al.^40^ reported that the IPDs determined by mixed-gender dyads did not significantly differ from those reported by same-gender dyads.

In our study, however, the results exhibited interactions with the pandemic stages. As depicted in Fig. 3, when encountering a male target in the pandemic stage, female participants consistently exhibited larger IPD. Zhou et al.^41^ noted that IPD between individuals was associated with perceptual judgments related to social grouping, with female participants tending to maintain a larger distance in mixed-gender dyads due to feelings of insecurity and shyness^42^. Interestingly, the shortest IPD occurred when male participants faced a female target wearing a mask (133.3 cm) during the pandemic stage. In the post-pandemic stage, the shortest IPD was observed when a female participant faced a masked female target (61.4 cm), as depicted in Fig. 4. This observation aligns with the interactive influence of participant gender on IPD between the pandemic stages, as indicated in Table 1.

### Mask wearing

While various factors significantly influenced IPD during the epidemic stage, only the mask-wearing variable continued to exert a significant effect on IPD in the post-pandemic stage (*p* < 0.001, Table 2). Research on the impact of mask-wearing on IPD during the SARS-CoV-2 pandemic consistently shows a reduction in IPD when individuals encounter a mask-wearing target^2,3,6,8,17^. Our findings align with these previous results. However, the current research stands out as one of the few studies that explore the impact of wearing masks on IPD in the post-epidemic stage. Although mask-wearing remains the most effective and convenient preventive measure to control the SARS-CoV-2 pandemic, it seems to have a negative impact on people's perception of IPD. Our analysis suggests that wearing masks might lead people to prefer a closer and potentially riskier IPD, aligning with the risk homeostasis theory proposed by Wilde^43^. Significantly, in the post-pandemic stage, we observed a notable decrease in the average IPD from 145.7 cm to 71.6 cm when participants encountered a masked target (Fig. 2C).

Our findings indicate that, even as the SARS-CoV-2 epidemic has slowed down, the influence of masks on IPD persists. However, the difference decreases from approximately 50 cm in the epidemic stage to 20 cm in the post-epidemic stage (Fig. 2C). In contrast to the epidemic stage, participant and target gender no longer influence IPD in this stage. One potential explanation for the enduring mask effect is that the absence of a mask mandate does not imply the complete disappearance of the virus. According to our observations, many Taiwanese have continued to wear masks in the post-epidemic stage, with nearly 80% of people still wearing masks in public places. Zhang et al.^44^ employed depth detection devices to analyze close contact behaviors in railway carriages and surrounding spaces. Their findings indicated that when all passengers wore N95 respirators and surgical masks, personal virus exposure via close contact could be reduced by approximately 94% and 52%, respectively. The presence of a mask created a subjective perception of increased safety, resulting in a reasonable reduction in IPD.

Another noteworthy phenomenon is that during the initial stage of the test, which coincided with the peak of the epidemic, all participants consistently wore masks. In the second stage of the test, despite the lifting of the mandatory mask-wearing requirement in Taiwan, we observed that 65% of the participants continued to wear masks throughout the test without any specific instructions. Whether participants' diverse inclinations to wear masks impact the determination of IPD warrants further examination.

### Other considerations

While prior research has indicated that encountering a vaccinated target also shortens IPD^8^, vaccination, unlike other preventive measures such as mask-wearing, aims to protect individuals from infection and reduce the risk of mortality after diagnosis^45–47^. This distinction may lead to misperceptions about the role of vaccines and pose challenges in the prevention and control of SARS-CoV-2 transmission^8^. In our study, the vaccination status of the target was not manipulated, and related interference was consequently disregarded. We noted that all participants in both stages had received the required vaccine doses. Additionally, it is crucial to recognize that IPD is influenced by the epidemic status, reflecting varying levels of people's alertness and resulting in changes in IPD. This consideration should be taken into account in future research on IPD-related studies.

## Strengths and limitations

This study compared the changes in IPD in Taiwan during different stages of the SARS-CoV-2 epidemic, with an interval of approximately one year. The investigation also explored the impact of whether the target wore a mask and the influence of different sex dyads on IPD. The study collected IPD data under identical experimental settings for both stages to minimize interference. Our findings carry significant implications for normal times and during different epidemics. Given that IPD has increased due to coronavirus pandemic precautions, impacting behavioral norms, the knowledge generated during this period has expanded and deepened. This understanding can serve as a reference for the application and formulation of IPD-related policies in the future. Moreover, the study reveals a notable reduction in perceived IPD for social proximity in the post-pandemic stage. This reduction may signify the successful socio-cultural adaptation of the young Taiwanese individuals in this study during the post-pandemic era.

This study has also several limitations. The use of an online survey was necessitated by the pandemic and for comparison purposes, potentially resulting in IPD data that might not precisely reflect real-life situations. Additionally, the study exclusively used blue surgical masks, which may not account for the potential impact of different types and colors of face masks on IPD perception. In the discussion, making direct comparisons between the results of various studies was challenging due to the varying degrees of SARS-CoV-2 transmission at the time of data collection, which could affect participant perceptions. Furthermore, this study involved two stages of IPD testing, and only one-third of the participants were duplicated. As a result, the test stage and participant gender variables were considered between-subject designs, which could potentially introduce some bias. Given these limitations, future research should explore the effects of different types and colors of face masks on IPD perception, as well as consider conducting IPD tests in more controlled real-life situations to improve the generalizability of the findings.

## Conclusions

This study primarily investigated how the perception of IPD among young Taiwanese individuals, both men and women, was influenced by different pandemic stages (the pandemic and post-pandemic periods), with a one-year interval between the two test stages. The study findings revealed that, during the post-pandemic stage, participants perceived an average IPD that was approximately 90 cm shorter than during the peak of the pandemic. Notably, gender-related variables, affecting IPD in both participants and targets, were significant during the pandemic stage but became insignificant in the post-pandemic stage, with only the mask variable retaining its influence. These results suggest that in Taiwan, the interpersonal disconnect and spatial isolation necessitated for pandemic prevention rapidly receded in the post-pandemic stage. This shift may indicate a collective yearning for the restoration of social relationships and a return to more typical social interactions.

## Data Availability

The datasets used and analyzed during the current study are available from the corresponding author upon reasonable request.
